# COPD after “Tabouna” Exposure: A Distinct Phenotype in Tunisian Women?

**DOI:** 10.3390/jcm12237424

**Published:** 2023-11-30

**Authors:** Besma Hamdi, Sabrine Louhaichi, Mohamed Aymen Jebali, Frédéric Schlemmer, Bernard Maitre, Agnes Hamzaoui

**Affiliations:** 1 Laboratory Research 19SP02 ‘Chronic Pathologies: From Genome to Management’, Department of Respiratory Diseases, Faculty of Medicine of Tunis, Tunis El Manar University, Tunis 1068, Tunisia; sabrine.louhaichi@fmt.utm.tn (S.L.); jebalimaymen@gmail.com (M.A.J.); agnes.hamzaoui@gmail.com (A.H.); 2 Department of Respiratory Diseases, B. Abderrahman Mami Hospital, Tunis 2080, Tunisia; 3 Department of Respiratory Diseases, Créteil Intercommunal Hospital Center, 94000 Paris, France; frederic.schlemmer@aphp.fr (F.S.); bm.maitre@gmail.com (B.M.)

**Keywords:** COPD, women, combustible biomass, phenotype, never smokers, lung function

## Abstract

Background: COPD due to exposure to combustible biomass is an increasingly recognized phenotype, particularly among women who use traditional ovens, known as ‘Tabouna’, for baking bread. This paper aims to investigate the clinical and functional characteristics of COPD in Tunisian female patients attributed to the use of ‘Tabouna’. Methods: A retrospective single-center cohort study was conducted on patients recruited from the Department of Respiratory Disease at A. Mami Hospital, who were diagnosed with COPD between January 2014 and December 2022. The diagnosis of COPD adhered to the standards defined in GOLD 2022. Results: Out of the 95 women included in the study, 48 (50.5%) were exposed to tobacco smoke, while 47 (49.5%) were exposed to the ‘Tabouna’. The median age was 70.4 ± 11.5 years, ranging from 40 to 95 years. Patients exposed to biomass were notably older, with a median age of 75.4 compared to 64.6 (*p* = 0.04). A significant association was observed between COPD and biomass smoke exposure, both in women residing in rural and urban areas (*p* = 0.006). The frequency of patients exposed to biomass with comorbidities was higher than in the group exposed to tobacco, but only hypertension showed statistically significant results (*p* = 0.01). Tobacco smoke induced more impairment in lung function than biomass in the group with FEV1 ≤ 30% (*p* = 0.04). Long-acting muscarinic antagonists were more commonly prescribed to smokers (*p* = 0.04). Serious complications such as chronic respiratory failure and intensive care admissions were similar in both groups (*p* = 0.8 and 0.4). Conclusions: COPD in women after exposure to the ‘Tabouna’ was observed in older patients and characterized by delayed diagnosis. Despite these clinical differences, poor COPD outcomes were similar in both groups.

## 1. Introduction

Chronic Obstructive Pulmonary Disease (COPD) is an increasingly common condition. For a long time, it has been predominantly considered a disease affecting men [1]. Female involvement not only remains underdiagnosed, but is also underrepresented in clinical studies, particularly among non-smokers. When presenting with identical symptoms, 75% of men would receive a COPD diagnosis, while the figure is only 50% for women. Although smoking is recognized as a major risk factor, exposure to biomass and indoor or outdoor pollution also plays a significant role in the development of COPD, particularly among women residing in low-income countries [2,3]. Data from the Global Initiative for Lung Disease suggests that between 17% and 38% of global COPD cases are observed in non-smokers [4,5].

**Figure 1:**
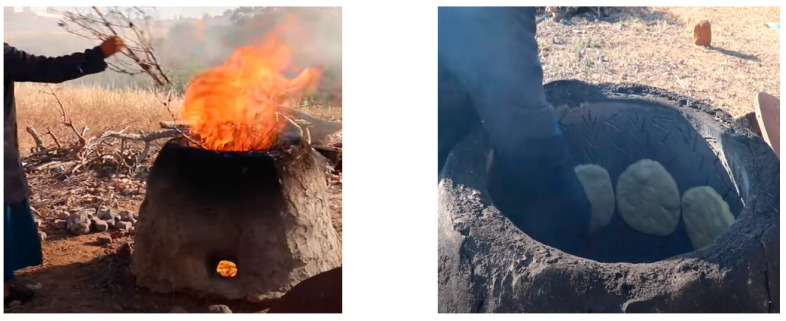
Tabouna is a traditional method used to bake bread in a mud oven using a wood fire.

The use of solid fuels, such as charcoal or firewood, for traditional baking represents a significant risk factor for COPD in women. In Tunisia, the preparation of ‘Tabouna’ bread is the most common source of exposure for women (Figure 1). This traditional method of baking bread exposes women to noxious agents, increasing their risk of developing lung diseases, particularly COPD. Cardiovascular diseases, including hypertension, atherosclerosis, and acute myocardial infarction, have also been reported. The clinical and functional presentations of COPD are characterized by heterogeneity. Exposure to smoking, air pollutants, biomass, and genetic factors appear to induce different inflammatory reactions, resulting in diverse clinical findings [6].

To assess the COPD phenotypes associated with specific exposures, it is essential to compare clinical findings between patients exposed to combustible biomass and those exposed to tobacco.

## 2. Methods

A retrospective single-center cohort study was carried out in the Respiratory Department B of Abderrahmen Mami Hospital. The medical records of patients diagnosed with chronic obstructive pulmonary disease between January 2014 and December 2022 were reviewed.

The eligible patients were aged over 40, had a history of exposure to either tobacco or biomass smoke, and had post-bronchodilator functional evidence of COPD, indicated by a forced expiratory volume in 1 s (FEV1)/forced vital capacity (FVC) ratio less than 70%.

Because of the specific nature of the exposure, all the patients were female. Patients with a history of asthma, bronchiectasis, lung malignancy, or interstitial lung disease, as well as those with a history of dual exposure (tobacco smoke and biomass), were excluded.

The medical records were thoroughly reviewed to gather demographic, clinical, laboratory and functional data. Spirometry values including (FEV1, FVC, FEV1/FVC) were collected and reported in accordance with the established standards.

**Figure 2:**
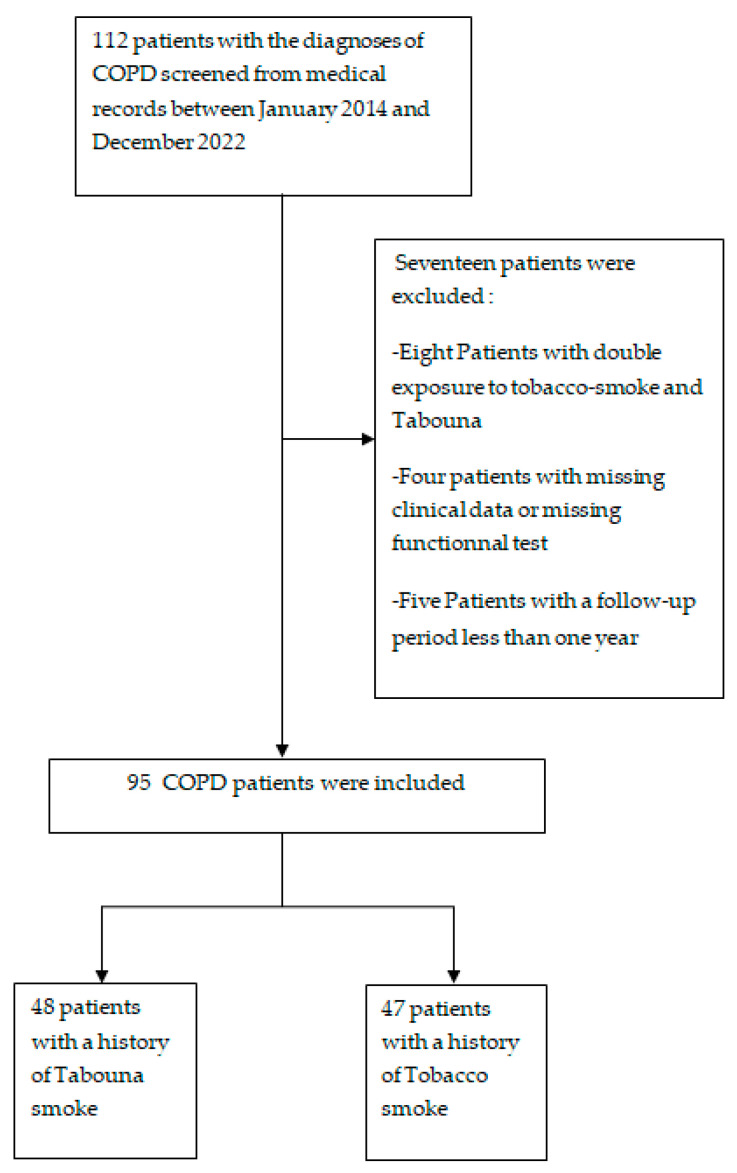
A flow chart of the study design.

We categorized two comparison groups according to the nature of the smoke exposure as illustrated in Figure 2.

Group 1: Biomass-smoke-exposed patients: this group included individuals who were exposed to daily biomass smoke for over a year and who had no history of smoking. The accumulated biomass smoke exposure index was determined by calculating the average number of hours spent using ‘Tabouna’ daily divided by the total number of years exposed.

Group 2: Controls: this group comprised patients with a history of exposure to tobacco smoke who were never exposed to biomass smoke.

The statistical analysis was performed using SPSS software version 25.0. Categorical variables were presented as numbers (percentages) and analyzed using the Chi-squared test or Fisher’s exact test. Continuous variables with normal distribution were expressed as mean ± standard deviation and analyzed using independent samples *t*-test, while those with skewed distribution were presented as median (Q1, Q3) and analyzed using the Mann–Whitney U test. A *p*-value of <0.05 was considered indicative of a significant association. For analyses, FEV1 and FVC were treated as continuous variables and modeled as % predicted FEV1 and % predicted FVC.

Written informed consent was not required due to the retrospective nature of the study and the use of anonymized data. The study design received approval from the Ethics Committee of Abderrahmen Mami Hospital

## 3. Results

Characteristics of the study population

We enrolled a total of 95 female patients in our study, comprising 48 individuals (50.5%) with a history of tobacco smoke exposure and 47 (49.5%) exposed to biomass smoke. Twenty-eight patients (59.5%) hailed from rural areas, while in the exposed to biomass group, the number of patients was 12 (25%) (*p* = 0.006).

The median age of the participants was 70.4 ± 11.5 years, with an age range spanning from 40 to 95 years. Notably, patients exposed to biomass smoke exhibited a higher median age of 75.4 years vs. 64.6 y, *p* = 0.04.

The prevalence of the most common comorbidities was as follows: obesity (45.2%), hypertension (37.9%), diabetes (18.1%), dyslipidemia (6.3%) and coronary artery disease (2.1%).

Notably, patients exposed to “Tabouna” smoke exhibited a significantly higher body mass index compared to smoking patients (31 kg/m² [18–54] versus 27 kg/m² [15–40], *p* < 0.001). Additionally, obesity and hypertension were more prevalent in this group of patients. Table 1 illustrates patients’ epidemiological data.

COPD outcomes

The mean duration of biomass exposure was 28 years, ranging between 15 and 53 years, with a mean accumulated biomass-burning smoke exposure index of 126 h per year.

Among the participants, twenty-five patients were still current smokers, with a mean smoking history of 38 pack years [ranging from 20 to 100]. The average follow-up period was 7.1 years [ranging from 1 to 30].

The median age at first diagnosis was 59 years old [43–77] versus 71 years old [53–85], *p* = 0.001.

The most common respiratory symptoms in both groups included exertional dyspnea (82%), cough (53.6%), sputum (36.8%) and wheezing (14.7%) as shown in Table 2.

The requirement for long-term oxygen therapy and/or long-term non-invasive ventilation was comparable in both groups, as shown in Table 2.

Pulmonary hypertension was seen in 16.8% of the total cohort study, without a significant difference between the two groups (16.6% versus 17%, *p* = 0.9).

**Figure 3:**
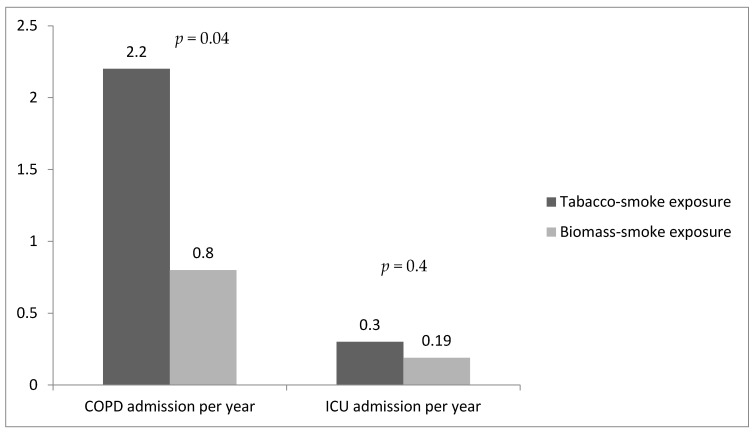
Total COPD admissions.

The median number of admissions for COPD acute events per patient per year was higher in the tobacco smoke group (*p* = 0.04), but no statistically significant difference was observed in the number of intensive care hospitalizations (Figure 3).

Pulmonary function:

At a baseline, the mean predicted FEV1 for the entire study population was 55% and the predicted FVC was 72%.There was no significant difference observed between the two groups regarding FEV1 % pred, FVC % pred or the FEV1/FVC ratio, both before and after the bronchodilator test (Table 3).

**Figure 4:**
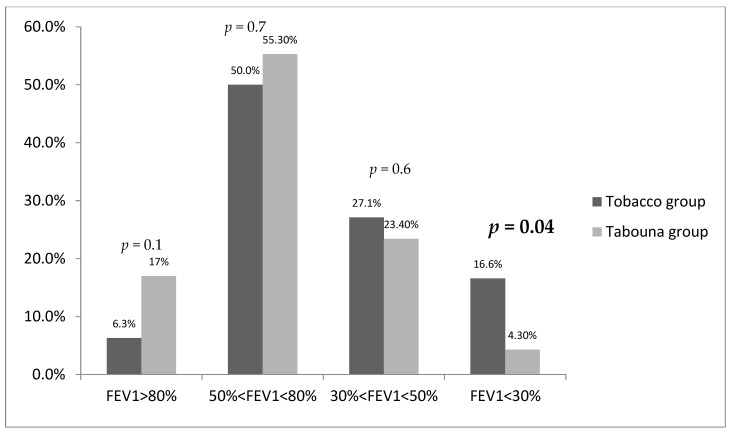
Patients’ distribution according to FEV1 value.

Patients with a history of tobacco exposure were more likely to have a FEV1 less than 30% (*p* = 0.04) (Figure 4).

Pharmacological treatment

All the patients with a history of biomass exposure were treated with long-acting β2-agonists (LABA). Notably, more patients in the tobacco group were prescribed long-acting muscarinic antagonists (LAMA) (*p* = 0.002). The prescription of inhaled corticosteroids (ICS) was similar in both groups (Table 4). 

## 4. Discussion

Exposure to biomass smoke is increasingly recognized as a significant risk factor for COPD in women. Through this comparative study between two groups of patients, one exposed to tobacco smoke and the other to “Tabouna” smoke, we were able to identify differences in the epidemiological profile of the disease. Patients exposed to “Tabouna” smoke were not only older than smokers, but also experienced a longer duration of symptoms before diagnosis and were more frequently afflicted with hypertension as a comorbidity. These data align with the recognized heterogeneity of the disease, which has been increasingly described as encompassing various etiological phenotypes. It is important to note that all the patients in our study were female, reflecting the specific exposure related to traditional bread cooking. Our study uncovered that a noteworthy proportion of women exposed to Tabouna smoke met the criteria of COPD as per GOLD 2022, exhibiting persistent respiratory symptoms and airflow limitations resulting from airway and alveolar lesions [7].

This finding was in line with reports from various countries including India, China and Canada, which suggest that women could be more susceptible to non-smoking risk factors associated with COPD [8,9,10].

In developing countries like Tunisia, cultural practices often lead women from rural areas to spend approximately five hours each day baking bread in a mud oven using a wood fire. WHO has estimated that women exposed to biomass smoke from such activities inhale an astonishing 25 million liters of polluted air. Multiple studies have shown that biomass exposure significantly increases the risk of COPD with an OR: 2.44 [1.79–3.33], both in subjects who smoke, OR: 4.39 [3.38–5.70] and non-smokers, OR: 2.55 [2.06–3.15] [11].

According to the WHO, more than 50% of the global population will face exposure to smoke generated from biomass combustion, with 90% of these exposures occurring in rural areas. This trend is expected to increase by 2030 [12].

In our current study, it is worth noting that the group exposed to biomass smoke included a higher number of patients from rural areas, with 28 patients (59.5%), compared to the tobacco group, which had 12 patients (25%) (*p* = 0.006).

The pathophysiology of COPD attributed to tobacco smoke has been extensively studied. It is characterized by a decline in expiratory airflow, emphysematous damage of the pulmonary parenchyma, and/or the narrowing and obliteration of the small peripheral airways [13].

In contrast, the pathophysiological alterations associated with exposure to biomass smoke have not been thoroughly explored. The limited available studies have been marked by heterogeneity in the research groups, and have focused on respiratory diseases in general [14]. Notably, ARSLAN et al. and KARA et al. demonstrated in their respective studies that biomass exposure tends to induce greater fibrosis and peribronchovascular thickening [15,16]. 

These studies primarily focused on atypical cases within the domain of respiratory diseases rather than specifically addressing COPD [14,15,16]. In a prospective study conducted in Mexico, the authors showed that COPD patients exposed to biomass exhibited a higher prevalence of TH2 cells compared to those exposed to tobacco smoke. This suggests the presence of distinct inflammatory responses [17]. Further prospective studies are essential to elucidate the mechanisms underlying Tabouna-induced COPD.

Our findings align with the anticipated range of mean ages, typically between 65 to 70 years, as observed in some international reviews of COPD attributed to organic dust exposure. Consistent with our clinical experience, our results reflect the aging of the population exposed to biomass smoke, delayed diagnosis, and the underestimation of symptoms in this patient group. These results are in line with the findings of studies conducted in many other countries that have employed similar methodologies to study biomass-induced COPD [18,19].

Comorbidities are highly prevalent in COPD and contribute to the increased severity of the disease, resulting in a diminished quality of life, and higher morbidity and mortality rates. However, the specific prevalence of comorbidities in COPD caused by biomass smoke, as opposed to COPD related to tobacco, remains underexplored and not well characterized.

The findings suggest that COPD resulting from exposure to “Tabouna” smoke predominantly affects women and is often accompanied by a higher burden of comorbidities. Notably, in our study, the comorbidities of high blood pressure and obesity were significantly associated with this form of COPD. The combustion of solid fuels, such as in the case of Tabouna, releases elevated levels of various gaseous pollutants, which may contribute to an increased risk of cardiovascular disease and mortality through mechanisms such as atherothrombosis [20].

The link between biomass exposure and the development of arterial hypertension may be influenced by shared risk factors related to lifestyle, environmental factors, and distinct systemic responses compared to tobacco exposure.

Obesity was another common risk factor within our female population, and it may, in part, account for the higher prevalence of arterial hypertension. While significant attention has been directed toward the impact of malnutrition on COPD outcomes, it appears that COPD associated with biomass exposure is more closely associated with a high body mass index (BMI). Some previous studies have indeed indicated that obesity may contribute to increased COPD morbidity [21,22].

We need to acknowledge several limitations in our study. Firstly, we did not investigate all potential comorbidities within our study population. Conditions like arrhythmias, pulmonary hypertension, peripheral arterial disease, anxiety, depression, and osteoporosis were not consistently documented in the patient records. These comorbidities, although common, could have provided valuable insights into the disease and may have influenced our study results [19,20,23,24]. The symptoms encompassed within the definition of COPD are not exclusive to the disease, and in our study, no significant differences were observed in terms of clinical symptoms and exposure. Dyspnea emerged as the most commonly reported symptom, aligning with findings from various other series [25]. Dyspnea, often representing a key reason for seeking medical evaluation, signals a decline in functional capacity. It is worth noting that in women with COPD who smoke, dyspnea scores are typically higher than those in men, and this has been linked to lower quality of life scores [26,27].

The onset of dyspnea is often delayed, which can contribute to a late diagnosis as individuals unconsciously adapt to exertional dyspnea by reducing their physical activity.

Both COPD groups exhibited abnormal spirometric parameters, including pre-bronchodilator (BD) FEV1, FVC, and FEV1/FVC, as well as post-BD FEV1, FVC, and FEV1/FVC. Notably, there were no significant differences between these parameters in the two groups. However, these parameters were relatively more favorable in the biomass-induced COPD group. Additionally, respiratory function was more compromised in the smoking-related COPD group, with statistically significant results being particularly notable in the subgroup with FEV1 ≤ 30%. Several studies have indicated that COPD due to biomass exposure affects the small airways and leads to increased air entrapment. These findings may account for the prevalence of clinical symptoms and the comparatively better respiratory function observed in this group of women [28,29].

Our study did not reveal any significant differences in terms of intensive care admissions. This aligns with findings from other studies demonstrating that the burden of respiratory exacerbations, disease progression, and the phenotypic characteristics of COPD are comparable in both smokers and non-smokers [8,30,31].

Thomsen et al. reported in the Copenhagen General Population Study that individuals with COPD who had never smoked had an increased risk of respiratory hospitalizations, but this was not associated with a higher risk of total mortality [32].

While international recommendations for COPD management are well-established and widely recognized [7], there remains a gap in our understanding of the management and effectiveness of pharmacotherapies for COPD induced by biomass exposure. To date, there have been relatively few clinical trials that specifically address the treatment of this condition [1,33,34].

In our series, all patients received varying therapeutic regimens based on factors such as the severity of the disease, the socioeconomic status of the patients, and their level of adherence to treatment. Notably, we observed that patients with COPD attributed to tobacco exposure were more commonly prescribed long-acting muscarinic antagonists (LAMAs) when compared to those with biomass-induced COPD.

This study has several limitations. Firstly, it has a retrospective design and a relatively small sample size, which might limit the statistical power of the findings. Additionally, the lack of inclusion of male patients, even in the control group, was a deliberate choice, as ‘Tabouna’ use is exclusive to women. However, the results of this study contribute to a better understanding of the clinical profile of COPD due to biomass exposure in the context of the lifestyle of Tunisian women from rural environments.

In conclusion, our study highlights the challenges in managing COPD among rural women, primarily due to difficulties in accessing healthcare. These findings underscore the importance of implementing early screening measures for women exposed to biomass smoke, with the goal of reducing morbidity and mortality within this patient group. It did bring up several key points and suggested steps to improve outcomes in the diagnosis of biomass-induced COPD. Nonetheless, further large-scale multicenter studies are warranted to provide more insights into the pathophysiological characteristics of COPD, secondary to this specific type of biomass exposure.

## Figures and Tables

**Table 1:** Patients’ epidemiological data.

	Tabouna Smoke (%) N = 47	Tobacco Smoke (%) N = 48	*p*-Value
**Age group (years old)**			
40–59 (%)	2 (4.3%)	9 (18.7%)	**0.02**
60–79 (%)	28 (59.5%)	33 (68.8%)	0.3
>80 (%)	17 (36.2%)	6 (12.5%)	**0.007**
Median age (years old)	75.4	64.6	**0.04**
**BMI (Kg/m**²)	**31**	**27**	**<0.001**
**Co-morbidities**			
Obesity	29 (61.7%)	14 (29.1%)	**0.001**
Hypertension	28 (59.6%)	8 (16.6%)	**0.01**
Diabetes	11 (23.4%)	6 (14.5%)	0.16
Dyslipidemia	5 (10.6%)	1 (2%)	0.08
Coronary disease	2 (4.2%)	0	0.14
**Age at first diagnosis (years old)**	71	59	**0.01**

**Table 2:** Clinical outcomes.

	Tabouna (%) N = 47	Tobacco (%) N = 48	*p*-Value
Exertional Dyspnea	36 (76.5%)	45(87%)	0.1
Cough	28 (59.5%)	23 (47.9%)	0.2
Sputum	18 (38.2%)	17 (35.4%)	0.7
Wheezing	8 (17%)	7 (14.5%)	0.7
Long-term oxygen	13 (27.6%)	14 (29.1%)	0.8
Long-term NIV *	2 (4.2%)	3 (6.2%)	0.6

* NIV: non-invasive ventilation.

**Table 3:** Pulmonary function according to the different groups of patients.

	Biomass (%) N = 47	Tobacco (%) N = 48	*p*-Value
Pre-bronchodilator			
FEV1 % pred	54%	48%	0.2
FVC % pred	72%	70%	0.8
FEV1/FVC ratio	60%	56%	0.6
Post-bronchodilator			
FEV1 % pred	61%	49%	0.1
FVC % pred	71%	72%	0.1
FEV1/FVC ratio	62%	56%	0.2

**Table 4:** Distribution according to pharmacological treatment.

	Total Patients	Biomass (%) N = 47	Tobacco (%) N = 48	*p*-Value
LABA	89 (93.6%)	47 (100%)	44 (91.6%)	0.04
LAMA	24 (25.2%)	4 (8.5%)	20 (41.6%)	0.002
ICS	42 (44.2%)	22 (46.8%)	20 (41.6%)	0.6
Theophyllin	2 (2.1%)	2 (4.2%)	0	0.1

## Data Availability

The data presented in this study are available from the corresponding author upon reasonable request.

## Abbreviations

COPD	Chronicobstructive lung disease
GOLD	Global initiative for chronic obstructive lung disease
FEV1	Forced expiratory volume in one second
FVC	Forced vital capacity
NIV	Non-invasive ventilation
ICU	Intensive care unit
LABA	Long-acting β2-agonists
LAMA	Long-acting anticholinergics
ICS	Inhaled corticosteroid
WHO	Word health organization
